# Holistic Thermo-Optical Design of Laminate Layers for Halide Perovskite Photovoltaic Windows

**DOI:** 10.1021/acsenergylett.4c02017

**Published:** 2024-11-11

**Authors:** Kevin J. Prince, Nicholas P. Irvin, Mirzo Mirzokarimov, Bryan A. Rosales, David T. Moore, Harvey L. Guthrey, Axel F. Palmstrom, Colin A. Wolden, Lance M. Wheeler

**Affiliations:** † National Renewable Energy Laboratory, Golden, Colorado 80401, United States; ‡ Department Chemical and Biological Engineering, Colorado School of Mines, Golden, Colorado 80401, United States

## Abstract

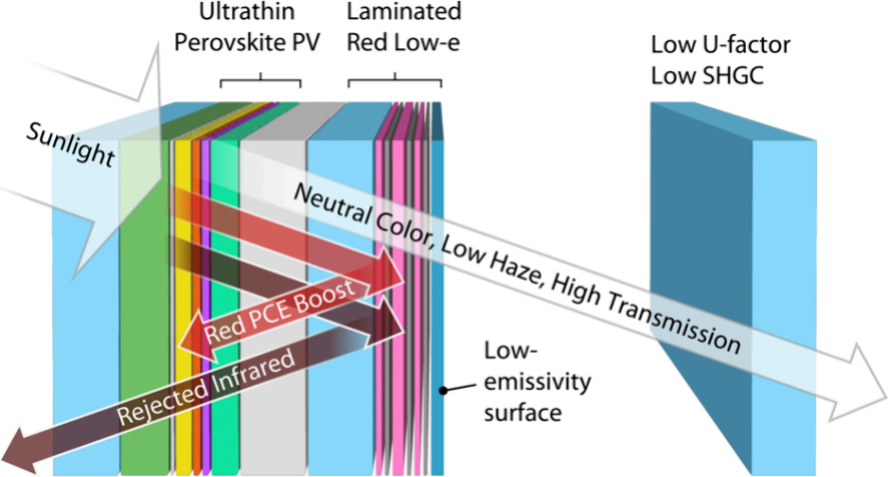

Though power conversion is an important metric for photovoltaic windows, it must be balanced with visible transmittance, aesthetics (color and haze), and thermal performance. Optical properties are often reported, but thermal performance is typically neglected entirely in photovoltaic window design. Here, we introduce the strategy of using laminate layers to improve the thermo-optical performance of perovskite-based photovoltaic insulating glass units. We design the laminates and insulating glass units by coupling a transfer matrix method optical model to a 1D heat transfer model. We validate our models with experimental fabrication of one-dimensional photonic crystal layers that are deposited on glass and laminated to the perovskite photovoltaic device. The holistic designs neutralize the inherent transmissive red color, and the “red low-e” design dramatically reduce emissivity of the glass surface to significantly improve thermal insulation and boost the photocurrent of the device.

During the energy transition from fossil-derived sources of energy to renewable sources, it is important to remember that *where* we generate energy is on the same order of importance as *how* we generate it. U.S. utility-scale electricity generation from source to load requires an average of 2.8 times more energy than onsite power generation.^1^ In 2010, buildings accounted for 32% of global energy use and 19% of energy-related greenhouse gas (GHG) emissions.^2^ In parallel, the cost of land and building materials has increased, whereas the cost of PV deployment has dropped by >85% in the past decade.^3^ Despite the obvious promise of building-integrated photovoltaics (BIPV) to leverage cheaper on-site energy and piggyback building materials cost, it is rare for buildings to utilize the many areas of the building surface beyond PV panels applied to the roof.

The increasing desire for architectural glass makes deployment of BIPV more difficult. Glass curtain walls have dominated high-rise building architecture since the 1950s despite low thermal performance.^4^ Since the 1980s, we have evolved from single-pane glazing to insulating glass units (IGUs) combined with low-emissivity (low-e) coatings to drastically improve the thermal performance of windows and decrease building energy usage.^5^ There is a significant opportunity to utilize the large glass surface areas around buildings to generate electricity using PV windows.^6^ PV windows are IGUs with semitransparent solar cells. Recent work suggests PV windows could cut energy use and CO_2_ emissions by 40% in highly glazed buildings compared to the use of standard double-paned windows.^7^

PV windows can be constructed from various PV technologies that we divide into two main categories based on the absorber used: (1) transparent, or wavelength-selective designs; and (2) semitransparent, or nonwavelength-selective designs.^8^ Wavelength-selective PV designs use excitonic absorption of organic materials in thin films^9,10^ or dyes^11^ to selectively absorb ultraviolet (UV) and near-infrared (IR) light. Nonwavelength-selective designs utilize prototypical semiconductor PV materials such as amorphous silicon (a-Si),^12^ cadmium telluride (CdTe),^13^ copper indium gallium selenide (CIGS),^14^ or metal halide perovskites (MHPs),^15,16^ which absorb visible light. To achieve high visible light transmittance, nonwavelength-selective absorbers must have a wide bandgap, which significantly limits efficiency,^17^ patterned to allow light through between the opaque areas,^18,19^ or be thinned down for semitransparency.^20^

To date, PV window research often takes a limited view of PV window design by focusing on the trade-off between PV power conversion and transmission of visible light.^8^ Though this is indeed a fundamental design parameter, other considerations including thermo-optical performance are often treated as secondary or scarcely studied from those developing new PV window technologies.^10^ PV windows will only see widespread deployment if they can achieve appreciable power conversion, in addition to architecturally acceptable optical properties and high thermal performance.

In this work, we discuss the complex design space that must be considered when developing a PV window and present a comprehensive design of MHP-based PV windows using thermo-optical modeling and validation with experiment. Our designs minimize changes to the MHP device stack and center on laminating secondary glass layers that are outfitted with one-dimensional photonic crystal layers that selectively reflect light to simultaneously balance architecturally acceptable aesthetics and boost photocurrent in the PV device. “Red low-e” laminates are further designed with reduced emissivity to dramatically improve thermal performance of the resulting IGUs. Our holistic design yields perovskite PV windows that balance the many factors needed for PV window performance by demonstrating high efficiency, tunable and architecturally acceptable aesthetics, and extraordinary thermal insulation.

We start with a general discussion of PV window performance metrics to establish language and concepts used in our results and provide researchers with an overview of the many design criteria that should be considered. The solar energy spectrum governs the design of PV windows and is best presented in two different forms to understand the balance of PV power conversion and thermo-optical performance— photon flux (*F*(E), Figure 1a) or spectral irradiance (*I*(E), Figure 1b) as a function of wavelength or energy. Photon flux is the number of photons, whereas spectral irradiance weights each photon by its energy.

**Figure 1:**
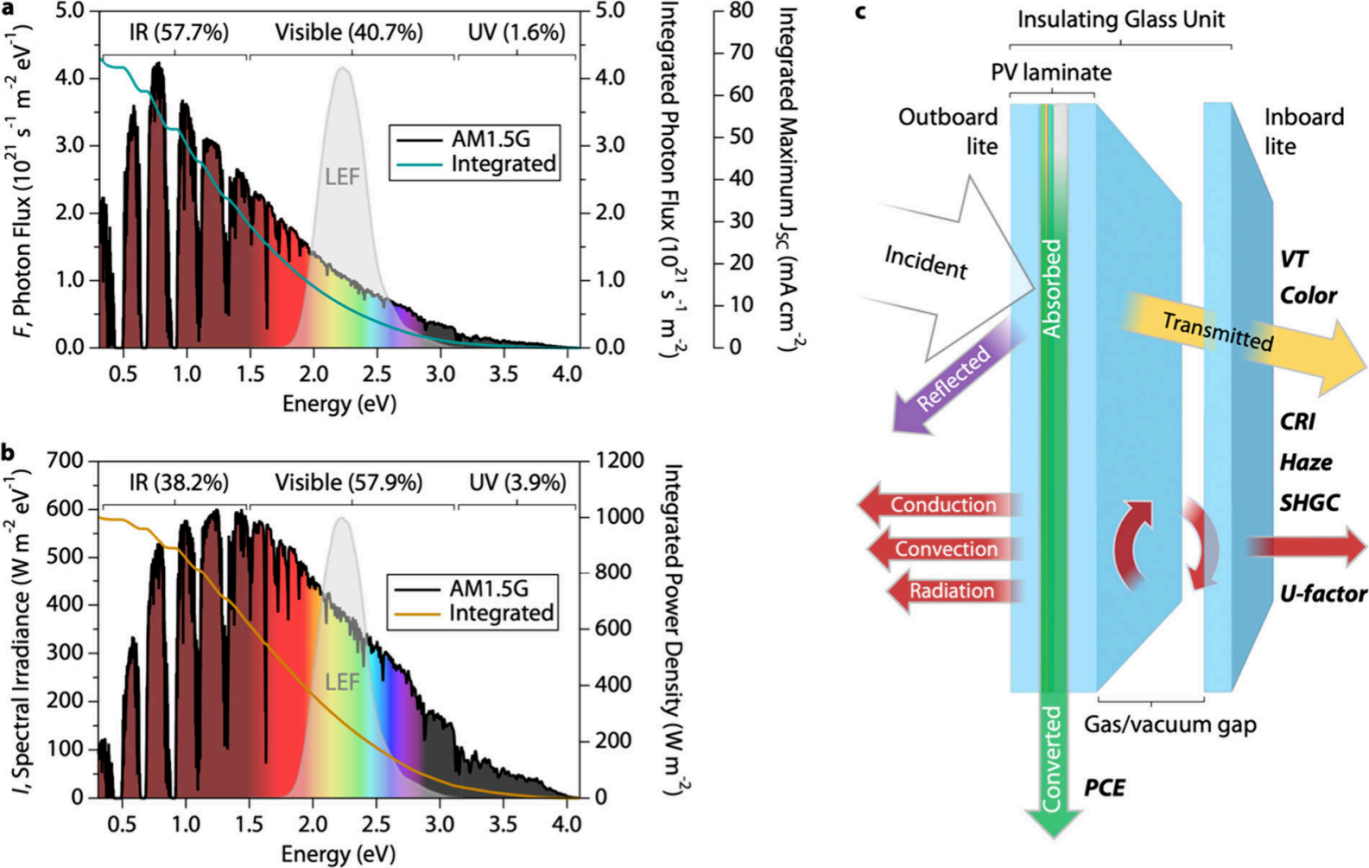
Designing PV Windows across the solar energy spectrum. (a) AM1.5 Global (AM1.5G) solar photon flux, integrated photon flux, and integrated maximum short-circuit current density (*J*_SC_) as a function of solar photon energy. (b) AM1.5G power flux (spectral irradiance) and integrated power density as a function of solar photon energy. Solar spectra are broken down into number (a) and energy (b) of photons in the ultraviolet (UV), visible (Vis), and infrared (IR) portions. LEF = luminous efficiency function. (c) Schematic of an insulating glass unit (IGU) with a PV laminate serving as the outboard lite. Energy flow is illustrated, with different performance metrics highlighted.

Power conversion efficiency (PCE) of a PV cell can be presented in terms of spectral power conversion (Table 1), where the short-circuit current density (*J*_*SC*_(*E*)) is a function of the energy-dependent solar photon flux (Figure 1a). Each absorbed photon generates an electron–hole pair; hence, *F*(E) dictates device output power rather than the spectral irradiance. Integration of the photon flux spectrum thus yields the maximum theoretical *J*_*SC*_(*E*) of a PV device by multiplying *F*(E) by the elementary charge (1.60217663 × 10^–19^ C).

**Table 1:** Overview of the Many Thermo-Optical PV and IGU Performance Metrics with Emphasis on Solar Spectrum Dependance and Measurable Spectra or Quantities Needed to Calculate Them^a^

	**Metric**	**Equation**	**Units**
1	Power conversion efficiency (PCE)		%

2a	Visible transmittance (VT)		-
2b	Visible light transmittance (VLT), Average visible transmittance (AVT)		%
3	Light utilization efficiency (LUE)		%
4	Haze		%
5	CIELAB Color	,	-
		,,	

6	Color rendering index (CRI)		-
		(	
7	Solar heat gain coefficient (SHGC)*		-
8	U-factor*	,	

a *q* is the elementary charge. EQE is the external quantum efficiency. The subscript *i* = *T* is for the glazing transmission spectrum, and *i* = *I* is for the solar spectral irradiance spectrum. x̅, y̅, and z̅ are the CIE XYZ standard observer color matching functions (Figure S2), *j* = 1-8 are the eight CIE test-color samples.^32^ All integrals are evaluated over the energy of the solar spectrum. *Effect of converted photons is not included in this equation.

Visible transmittance (VT) is also determined using *F*(E) and is defined as the sensitivity-weighted ratio of transmitted to incident photons in the visible spectrum. Spectral sensitivity in the human eye is defined by the luminous efficiency function (LEF) (Table 1, Figure 1a,b).^21^ The LEF is also often called the eye response function or photopic eye response. Human eyes are most sensitive to green light (555 nm), but sensitivity extends from 390 nm out to 830 nm (Figure S1). Based on this range, the infrared (IR) portion of the spectrum is the richest in photons, representing 57.7% of the solar spectrum, whereas the visible and ultraviolet (UV) portions encompass 40.7% and 1.6%, respectively. VT is used by the glazing industry and is expressed as a value between zero and unity. Average visible transmittance (AVT) and visible light transmittance (VLT) are also often found in the literature, which have the same definition as VT but expressed as a percentage (Table 1).

High VT is often strived for to accommodate aesthetic and occupant preference. We have also previously studied the impact of VT on the trade-off between power generated by the PV window and increased lighting power consumption needed to accommodate less natural light. PV windows with relatively high VT (VT > 0.4) do not change lighting power consumed by the building. Even for extremely low VT case (VT < 0.05), the generated energy is roughly an order of magnitude higher than the increased energy consumption due to artificial lighting.^7^

PV windows must be designed to have high PCE balanced with acceptable VT. Review articles extensively cover this topic for different types of PV absorber technologies.^8,22^ Light utilization efficiency (LUE), defined as the product of PCE and VLT, is a recently developed and intuitive metric for maximizing optical and electrical performance (Table 1).^8^ However, LUE does not capture the importance of haze and color for vision glazing—architectural glass used for view of the outdoors.

High VT is meaningful only when haze is low and color is aesthetically acceptable. Haze is defined as the ratio of diffuse transmittance (*T*_d_) to total transmittance (*T*_t_) (Table 1). An increase in light scattering through a material will increase haze. ASTM D1003-21 defines the haze of a material in qualitative terms as “the reduction in contrast of objects viewed through it.”^23^ The human eye is extremely sensitive to changes in haze, as observers can detect changes in haze of <1% through glass.^24^ Since architectural glass is nearly haze-free, any deviation >1% haze from conventional glass will be noticed by building occupants.

Color can be quantified using various scales, but the CIELAB scale has become standard for architectural glazing, which quantifies color based on “lightness” (L*) and chromaticity (a*,b*) from calculations performed on the tristimulus values *X*_*i*_, *Y*_*i*_, and *Z*_*i*_ (Table 1). Though color tunability has often been touted in the literature for semitransparent PV and may find niche applications,^25^ architects far-and-away prefer neutral gray coloration, followed by blue and green to a lesser extent.^26^ With the one exception of bronze, which has seen bouts of popularity, red or yellow hues have never created the popularity needed to warrant commercial production.^26^

The most widely used metric for vision glazing to quantify color neutrality, and recommended for PV glazing,^27^ is the color rendering index (CRI). CRI is the geometric difference in the chromaticity and “lightness” (Δ*E*_i_^*^) of a sample illuminated with a reference source (AM1.5G for windows). The difference is based on the average of eight Commission Internationale d’eclairage (CIE) standard test-color samples illuminated with the AM1.5G through the window (Table 1).^28^ CRI is not a percentage but a number between 0 and 100, where a CRI of >90 is considered color neutral and aesthetically acceptable for most architectural applications. Despite how the human eye functions, spectral irradiance, rather than *F*(E), is used to calculate the CRI based on an established formalism. A detailed PV window-specific discussion and useful spreadsheet resource on color and CRI was recently published by Lunt et al.^27^

In contrast to PCE and VT, thermal performance is based on control of energy transfer to and from the building and is best analyzed with spectral irradiance (*I*(E)), where each photon is weighted by its energy (Figure 1b). Weighting by photon energy shifts the peak of the spectrum toward the visible portion, where 57.9% of the total energy is encompassed (Figure 1b). Light management outside the visible region is often neglected research in new PV window technologies, but it is critical for thermal performance. High thermal performance requires the PV laminate to be integrated into an IGU composed of outboard and inboard lites (panes of glass or multiple panes laminated together), insulating spacers, gas or vacuum gaps, and thin-film coatings (Figure 1c). Though several permutations have been explored in the literature,^29^ commercially available PV windows are fabricated with an outboard PV laminate composed of PV devices laminated between two pieces of glass. The PV laminate serves as the outboard lite of the IGU.

Modern non-PV IGUs are always designed with low-emissivity (low-e) coatings to improve thermal performance.^30^ Low-e coatings dramatically impact the solar-heat-gain-coefficient (SHGC), defined as the fraction of solar energy transferred into the building due to two factors: (1) transmitted photon power (product of spectral irradiance and transmission, *I*(E)*T*(*E*)) and (2) the fraction of the power absorbed by the IGU and subsequently transferred into the building through conduction, convection, or radiation through the relationship *n*(*E*)*I*(E)*A*(*E*)), where *n*(*E*) is the fraction directed inside and *I*(E)*A*(*E*) is the absorbed power as a function of photon energy (Table 1).^31^ Low-e coatings reduce *I*(E)*T*(*E*) by reflecting light and reduce *n*(*E*) by minimizing radiative heat transfer from, for instance, a high-temperature outboard PV laminate to the building interior.

It is important to emphasize two points about low-e coatings: (1) Emissivity is a surface property, so low-e coatings cannot be buried in a laminate and must face the gas or vacuum gap of an IGU to reduce heat transfer through the IGU. (2) Low-e coatings are typically designed to reflect infrared wavelengths of light (38.2% of the energy in the solar spectrum), but visible photons also contribute to SHGC as the most energetic part of the spectrum (57.9%, Figure 1b).

U-factor, also called U-value, is the overall heat transfer coefficient of an IGU in units of W m^–2^ K^–1^ and should be minimized for high thermal performance. All three modes of heat transfer, conduction, convection, and radiation, must be targeted to reduce U-factors. U-factor is a function of the thermal resistances of each part of the IGU where *h*_e_ is the heat transfer coefficient of the exterior surface, *h*_i_ is the coefficient of the interior surface, and *h*_t_ is the coefficient of the IGU itself (Table 1).^33^*h*_t_ is determined by a combination of thermal resistance in series and parallel, where *k* the number of gaps, *l* is the number of solid layers, *d*_m_ is the thickness of each solid material in the lite (glass, EVA, etc.), and *r*_m_ is the thermal resistivity of the material.  is the convection coefficient in the gas-filled gap, where *N*_u_ is the Nusselt number, *K* the gas thermal conductivity, and *s* is the width of the gap. Spacing of the gas gap is typically *s* = 12 mm to eliminate convective heat transfer and force conduction through the gas between lites.

U-factor is thus reduced by exchanging air for low-*K* gases (e.g., Ar and Xe).  is the radiation heat transfer coefficient, where σ is the Stefan–Boltzmann constant, ε_1_ and ε_2_ are emissivities of either side of the gap corrected for the mean absolute temperature *T* of the gas space. Low emissivity is critical to a low *h*_r_ and low overall U-factor. Current high-performance IGUs couple low thermal conductivity gas with low-e films to minimize conduction and shut down convection and radiation. For reference, insulated and opaque walls have U-factors of 0.1–0.2 W m^–2^ K^–1^,^34^ whereas code-compliant double-pane IGUs with a single low-e layer have U-factors of 1–1.5 W m^–2^ K^–1^ at center of glass.^7^ Superinsulating IGUs are fabricated by removing gas conduction entirely by evacuating the gas in the gap between lites to form vacuum insulating glazing (VIG). VIG units with two vacuum gaps and four low-e coatings have shown U-factors as low as 0.5–0.8 W m^–2^ K^–1^.^35^

Though beyond the scope of this work, we note that the complex design space of PV windows could be expanded beyond thermo-optical performance. Economics are paramount in a risk-averse market like building construction. Thin-film PV offers a unique add-on to IGUs that will already be designed into a building. Module efficiency and durability dramatically impact the economics of deployment and must be maximized.^36^ From a manufacturing perspective, glass is the highest-cost component of thin-film solar panels in terms of money, energy, and carbon.^37,38^ Glass also constitutes the highest mass component of the module, with PV-specific layers contributing negligibly (<1%) to the mass of the product.^39^ The weight of a PV window should not be considerably different from conventional glazing and should not impact installation costs. Since windows are already going into the building, the largest driver in monetary cost for building is added electrical integration, which will decrease as market penetration increases.^40^ Finally, more glazing in a building exterior leads to poor acoustic performance of the building, allowing unwelcome outside noise into dwellings and places of work. Laminated exterior lites, with similar construction to a glass–glass module, are already used to significantly dampen sound compared with single-pane lites in IGUs, so PV IGUs should generally offer better sound protection. Recent work shows this strategy works in VIG units as well.^41^

There are several ways to form semitransparent nonwavelength-selective solar cells for vision glazing. Selective-area transmission is the most common commercial technology, which uses crystalline silicon PV by spacing cells in a glass–glass package to allow light between cells. More recent work on Si^42^ and MHP^43^ absorbers demonstrated microscale selective area transmission using micropatterning (etching or laser ablation), and MHP materials have been patterned using dewetting^44^ and templating.^22^ Microscale patterning leads to unacceptable haze (>1%) for vision glazing in architectural applications. Though low-haze nanoscale patterning has been demonstrated,^45^ the most common method is to thin the absorber.

Here, we formed tunable thickness semitransparent PV devices using triple-cation, double-halide MHP films, where the film thickness and transparency were tuned by adjusting the molarity of the MHP precursor solution. The solution was composed of 15.4 mg of CsI, 22.4 mg of MABr, 73.4 mg of PbBr_2_, 172 mg of FAI, and 507 mg of PbI_2_ dissolved in 1 mL of 9:1 dimethylformamide:n-methylpyrrolidone. During the spin process, nitrogen flow was controlled over the spinning substrate using an automated system through a quench nozzle to control film formation kinetics. Conventional MHP PV devices generally use ∼1.3 M precursor solutions, which produce ∼500 nm-thick films and appear black due to absorbing most photons across the visible spectrum.

Thinner and more transparent films are formed by simply diluting the precursor solution under the same conditions. Profilometry confirms that the lowest concentration precursor solutions (0.14 M) yield compact films with thicknesses between 30 and 35 nm (Figure S3). Thinner MHP films have a natural red-yellow color due to being more transmissive to red wavelengths compared to the blue and green (Figure 2a). Transmission steadily increases in the red wavelength region of the visible spectrum, as we dilute the precursor concentration to form thinner MHP films (Figure 2a). The sloped transmission profiles arise from the absorption coefficient increasing with decreasing wavelength, as nonselective semiconductor materials have an expanding density of states above the conduction band edge, increasing the probability of absorption of higher energy photons. All nonwavelength-selective semiconductor materials exhibit the same thinning behavior, including methylammonium lead iodide, Si, CdTe, CIGS, and III–V materials (Figure S4). VT increases with a decreasing MHP precursor concentration and film thickness (Figure 2b). Conventional thick MHP films exhibit VT = 0.03 (Figure 2c), whereas the thinnest MHP films exhibit VT = 0.60 (Figure 2c).

**Figure 2:**
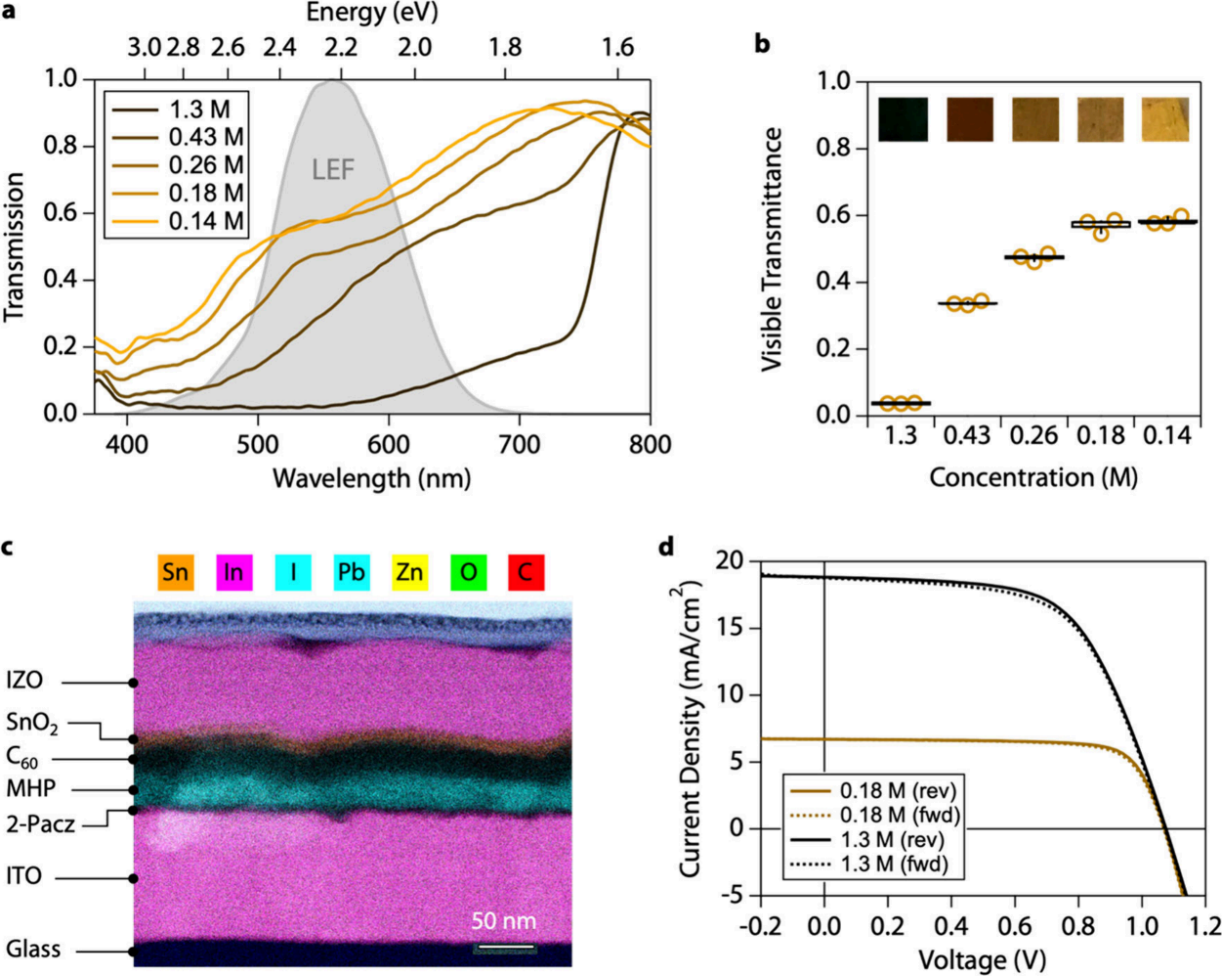
Ultrathin semitransparent metal halide perovskite PV devices. (a) Transmission curves over the luminous efficiency function (LEF). (b) Visible transmittance (VT) of metal halide perovskite (MHP) films with varying thickness controlled by varying the perovskite precursor solution concentration. (c) Scanning transmission electron microscopy (STEM) image overlaid with an elemental map produced with energy-dispersive X-ray spectroscopy (EDS). The topmost layer is tungsten to mitigate damage from incident Ga^+^ ions during the sample preparation. (d) Current–voltage scans of representative PV devices with thicknesses of 550 nm (1.3 M) and 35 nm (0.18 M).

Efficient semitransparent PV devices are completed by combining MHP films with electron- and hole-selective contacts optimized for high VT and limited parasitic blue photon absorption. We used [2-(9H-Carbazol-9-yl)ethyl]phosphonic acid (2PACz) self-assembled monolayers coated onto indium tin oxide (ITO) as the hole-selective contact because it maximizes VT with negligible absorption (Figure S5). 2PACz-functionalized ITO and other carbazole monolayers have emerged as favorable hole-selective contacts in MHP PV, particularly in perovskite-Si tandem solar cells due to fast hole extraction and minimizing nonradiative recombination at the hole-selective interface.^46^

For the electron selective contact, we evaporated C_60_ for efficient electron extraction and paired it with tin oxide (SnO_*x*_) deposited by atomic layer deposition (ALD) as a protective buffer layer for subsequent indium zinc oxide (IZO) sputtering (Figure 2d). Decreasing the C_60_ thickness from 30 to 15 nm and ALD SnO_*x*_ thickness from 35 to 15 nm increases transmission across the visible spectrum and decreases blue absorption (Figure S6). We also thinned the sputtered IZO to maximize the transparency (Figure S7). Thickness and composition of each layer in the ultrathin MHP device are apparent from the cross-sectional scanning electron microscopy (STEM) coupled with energy-dispersive X-ray spectroscopy (EDS, Figure 2c). The entire semitransparent MHP device stack is <300 nm thick.

The ultrathin MHP devices do not exhibit shunting despite an absorber thickness <50 nm. The thick and thin absorbers yielded devices with PCE as high as 12.2% and 5.3%, respectively. Thicker absorbers have higher efficiencies due to higher photon absorption, thus higher *J*_SC_, but fill factor was limited by high sheet resistance (70.5 ± 13.6 Ω/□) in the 80 nm IZO layer. Thinner absorbers have higher fill factors because lower photon absorption reduces short circuit current (*I*_SC_), which leads to less current and resistance losses in the IZO. The dependence of fill factor on series resistance can be approximated with FF = FF_0_(1–*R*_S_)*I*_SC_*/V*_OC_, where FF_0_ is the fill factor without series resistance.^47,48^ For a constant series resistance, the fill factor is linearly proportional to the short circuit current.

Current PV windows are composed of glass–glass laminates as the outboard lite of an IGU. MHP PV will need similar laminate packages to achieve commercially viable reliability by mitigating moisture ingress and preventing volatilization of MHP components under stress.^49^ To balance the transmitted color to neutral gray, we designed one-dimensional photonic crystals (also called dielectric mirrors or distributed Bragg reflectors) that are deposited on glass that is subsequently laminated to MHP PV devices.

Selective reflection in the Bragg reflector occurs through thin-film interference achieved with layers of alternating high refractive index (*n*_H_) material with thickness (*d*_H_) and low refractive index (*n*_L_) material with thickness (*d*_L_). At normal incidence, constructive interference occurs when the Bragg condition is met to yield a peak reflection at λ_Bragg_: *n*_L_*d*_L_ = *n*_H_*d*_*H*_ = λ_Bragg_/4. The intensity of the reflection (*R*_i_) is a power-law function of the number of pairs of Bragg layers, *N* where *R*_i_ = 1–4(*n*_L_/*n*_H_)^*N*^.

We used *PVwindow*, an open-sourced transfer matrix method (TMM) software developed by our group, to optically model and design our Bragg reflectors with existing optical data (Table S1).^50^ We chose alternating layers of MgF_2_ (*n*_L_, *d*_L_ = 118 nm) and MoO_3_ (*n*_H_, *d*_H_ = 75 nm) to selectively reflect red photons (Figure 3a). The Bragg reflectors were deposited via electron-beam and thermal evaporation onto a separate glass substrate and laminated to the ultrathin MHP device rather than deposited directly on top (Figure 3a).^25^ This strategy allows for independent design and development of the MHP device and packaging of laminate layers to yield a haze-free, color-neutral MHP PV laminate.

**Figure 3:**
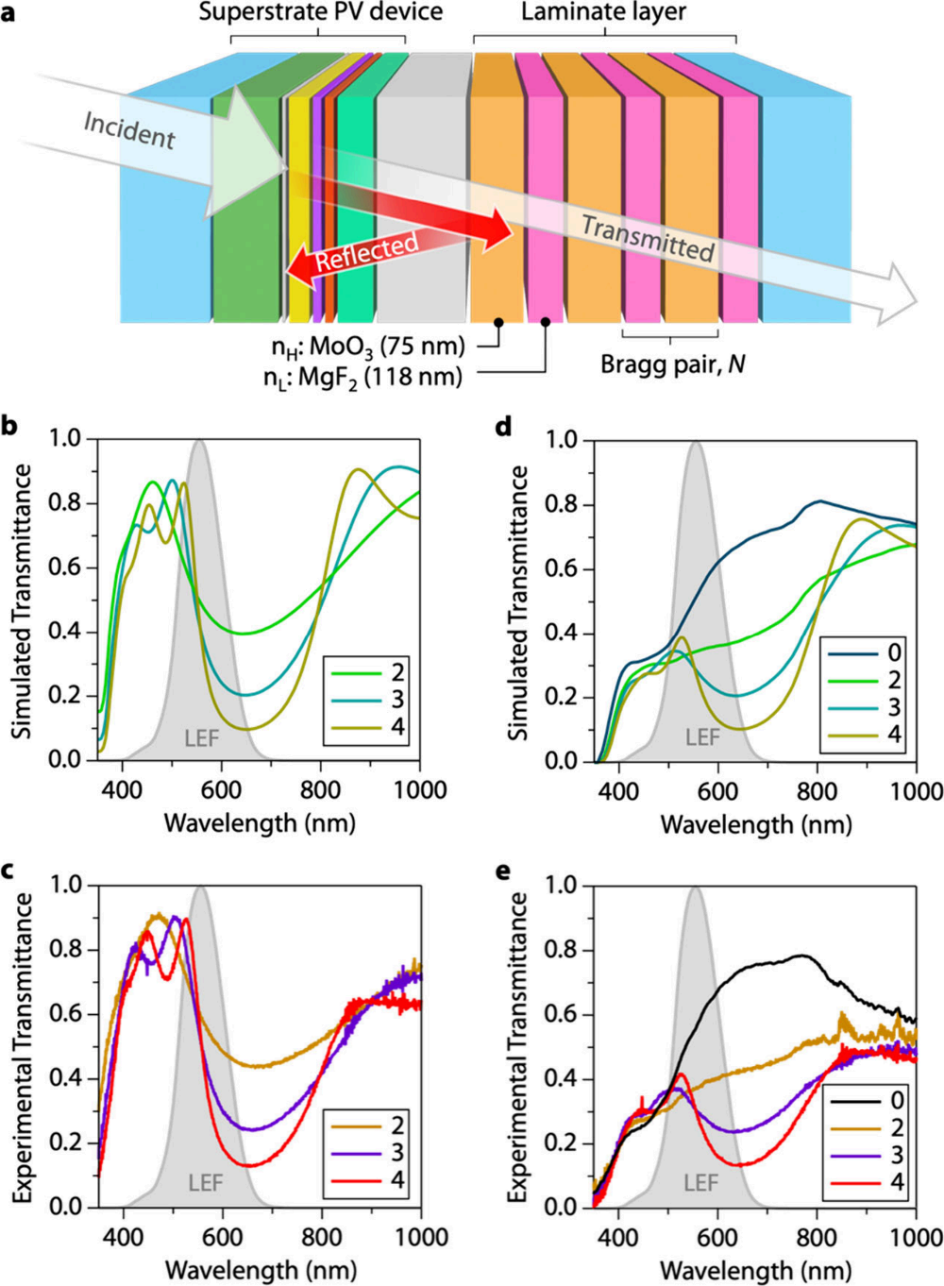
Fabrication and simulation of Bragg reflector laminate layers. (a) Diagram of MHP device laminated to a Bragg reflector laminate layer. Gray layer is an optical coupling layer. For simulation, ethylene vinyl acetate (EVA) was used, whereas a custom epoxy was used experimentally. (b) Simulated and (c) experimental transmission spectra of Bragg reflector laminate layers with *N* = 2, 3, and 4 Bragg pairs. (d) Simulated and (e) experimental transmission spectra of a Bragg reflector laminate layer coupled to a MHP PV device. *N* = 0 corresponds to the device with a 35 nm-thick (0.18 M) MHP layer with glass laminated to it without a Bragg reflector. LEF = luminous efficiency function.

The transmission spectra of our optically modeled Bragg reflectors (Figure 3b) match closely to those of our experimental laminate layer films (Figure 3c). For all films (*N* = 2–4), the reflection spectra peak at ∼625 nm with high transmission at bluer wavelengths. The transmission peak extends across the LEF function with a slope that is dependent on *N*. Reflection intensity and steepness of the overlapping reflection spectrum increase with *N*. Films with *N* = 2,3, and 4 Bragg pairs have a transmissive blue appearance and appear red when observing reflection. A sharp onset in the UV is maintained regardless of which Bragg reflector is integrated, indicating that the PV device stack will be protective against human-harmful wavelengths—an important consideration for window applications.

Transmission spectra of the unlaminated MHP device show good agreement between simulation (*N* = 0, Figure 3d) and experiment (*N* = 0, Figure 3e) with a sharp absorption onset at ∼380 nm, flat transmission between 380 and 450 nm, and increasing transmission beyond 450 nm. By incorporating a thick, optically incoherent layer as an optical coupling layer, we coupled the Bragg reflector laminate layer to the semitransparent PV device. Experimental data are nicely reproduced from the TMM simulation for *N* = 2–4 Bragg reflectors coupled to the device. For simulation, the coupling layer was 450-μm ethylene vinyl acetate (EVA). EVA is widely used in the Si solar industry to encapsulate solar cells onto glass sheets and in the laminated glass industry. Encapsulants such as EVA are typically between 300 and 1000 μm.^51^ Experimentally, we used a custom epoxy that cures at room temperature (methods) to avoid any degradation issues with the vacuum lamination needed for industry-standard encapsulants. The coupling layer is thick enough that there are no interference effects. EVA and the epoxy both exhibit high transmission at wavelengths absorbed by the cell, so the different materials will not significantly affect the simulation reproducing experiment.

Increasing the number of Bragg pairs of alternating MgF_2_ and MoO_3_ layers yields enhanced reflection of red photons, which monotonically decreases the VT (Figure 4a). Our simulation results agree well with our experimental results. For the simulated and experimental data, the spectrum of 35 nm-thick (0.18 M) MHP devices largely mirror the *N* = 2 Bragg reflector and intuitively yields a flattened transmission curve across the LEF function when combined into a laminate, which indicates a neutral color. The chromaticity of the laminated devices shifts from (a*, b*) = (9.19, 21.52) for *N* = 0 to (1.12, 6.93) for *N* = 2 based on the CIELAB chromaticity space (Figure 4b). Increasing *N* trends toward more negative values of a* (greenish) and negative values of b* (bluish), which are more aesthetically acceptable than the yellow/red coloration of the original device. CRI of the laminated MHP devices peak at 93 for *N* = 2 and decreases with increasing *N* (Figure 4c).

**Figure 4:**
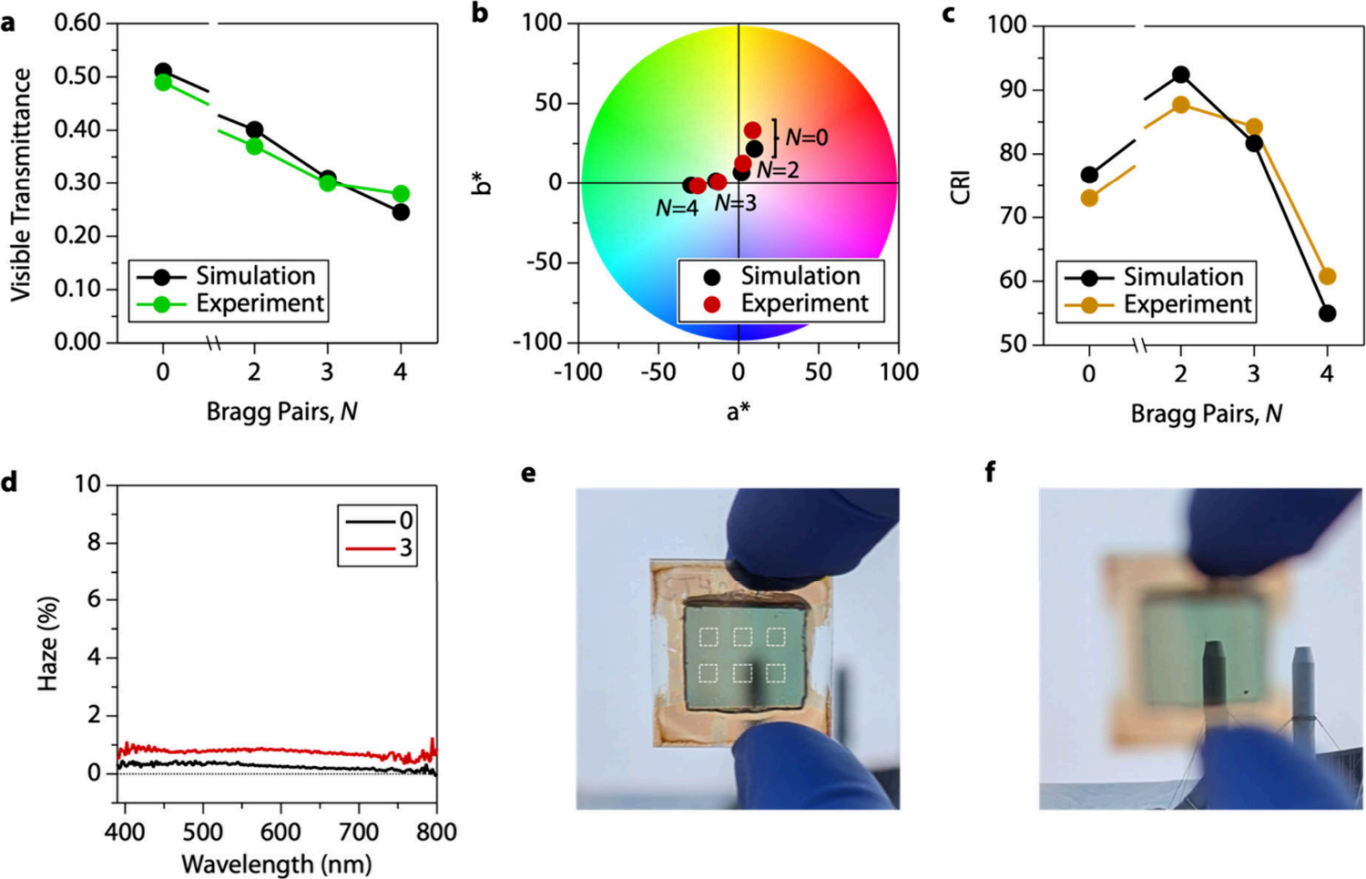
Optical properties of Bragg reflector laminated to an ultrathin MHP device. (a) Visible transmittance, (b) CIELAB chromaticity, and (c) CRI as a function of number of Bragg pairs, comparing simulation to experimental values. Color wheel in (b) adapted from ref (53). (d) Haze as a function of wavelength for ultrathin MHP PV devices (*N* = 0) and the same device laminated with an *N* = 3 Bragg reflector. Photographs of MHP devices laminated with a *N* = 3 Bragg reflector focusing on the device (e) and through the device to an exhaust tower ∼50 m in the distance (f) to illustrate clarity and lack of haze. Dashed white boxes in (e) indicate active areas of six pixels used to evaluate optical and electrical properties.

Haze is measured to be <1% across the visible spectrum for unlaminated devices (Figure 4d). Photographs of our laminated devices have a noticeable color change relative to unlaminated cells without introducing significant haze, where laminates maintain haze <1% (Figure 4e). Haze >1% will noticeably blur objects viewed through the window.^52^ A photograph of the same cell focused on an exhaust tower ∼50 m in the distance shows the same clarity as the adjacent exhaust tower viewed without the cell (Figure 4f).

In addition to balancing the transmissive color without introducing haze, Bragg reflectors reflect red photons back into the PV device to boost current density and PCE. Experimentally, *J*_sc_ monotonically increases as the number of Bragg pairs is increased from *N* = 0 to *N* = 4 (Figure 5a). We expand on optical simulations using *PVwindow* by extracting the absorptance spectrum of the MHP layer to perform detailed balance calculations based on the number of absorbed photons to determine theoretical maxima for *J*_sc_, open-circuit voltage (*V*_oc_), fill factor (FF), and spectral PCE (Methods). We assume unity internal quantum efficiency (IQE) for all *PVwindow* simulations, which circumvents the need to select arbitrary values for the material lifetime or contact selectivity and resistivity. The simulations cover the ideal limit, as the IQE is less than unity within experiments. The effect of lower IQE mainly lowers PCE. Simulated spectral PCE show the increased PCE is due to increased energy conversion at redder wavelengths close the MHP band gap (Figure 5b).

**Figure 5:**
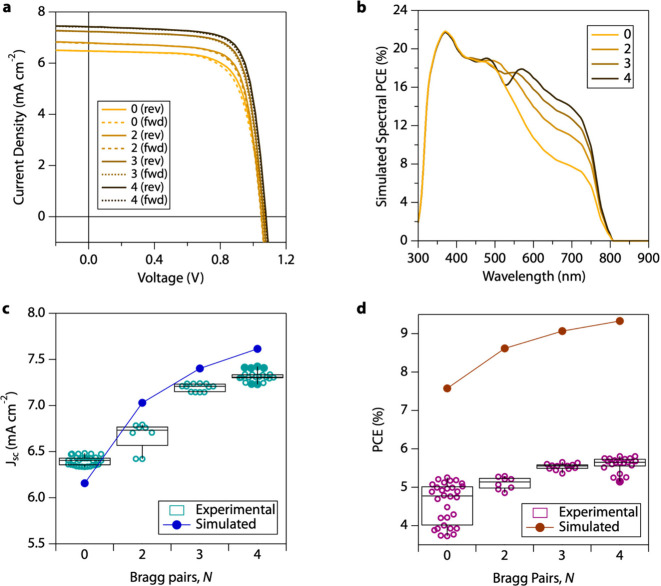
Photovoltaic performance of MHP PV laminates. (a) Experimental forward (for) and reverse (rev) current density−voltage scans of MHP PV laminates with *N* = 0, 2, 3, and 4 Bragg pairs. (b) Simulated spectral PCE of MHP PV laminates with *N* = 0, 2, 3, and 4 Bragg pairs. (c) Experimental and simulated short-circuit current density and (d) power conversion efficiency as functions of the number of Bragg pairs laminated to the cell. Box and whisker plots of the experimental data is based on the Tukey method where whiskers extend to the minimum and maximum values, and the box representing the interquartile range.

Increased conversion at a redder wavelength is due to increased absorption in that region of the spectrum, which increased photocurrent from the device. Our TMM model coupled to detailed balance is validated by experimentally observed enhancement in *J*_SC_. Our simulation predicts an increased *J*_SC_ from 6.15 mA cm^–2^ (*N* = 0) to 7.61 mA cm^–2^ (*N* = 4), a 23.7% boost, whereas our experimental data shows as an increased *J*_SC_ from 6.39 ± 0.15 mA cm^–2^ (*N* = 0) to 7.35 ± 0.25 mA cm^–2^ (*N* = 4), a 15% boost (Figure 5c). The boost in the photocurrent can also be observed in the PCE (Figure 5d). Though the experimental PCEs are lower by ∼3.5%, PCE values follow the expected trend from simulation, indicating that our devices lack fill factor and *V*_oc_ rather than *J*_sc_. The close match between modeled and experimental data demonstrates the efficacy of the *PVwindow* software and the applicability of further design. Other researchers and design teams could use this software as a tool to design similar one-dimensional PV window systems. The Bragg reflectors could be designed to adjust the color to a variety of colors,^54^ or better optimization algorithms could lead to more color-neutral designs that maximize photocurrent enhancement.

One-dimensional photonic crystals for color tuning of thin-film PV windows are now well established in the literature.^12,55,56^ Our laminate approach is distinct in that it decouples color balance from device processing, enabling simpler deployment and mix-and-match options for different colors and VTs. Here we also expand the functionality of the laminate layer by designing the photonic crystal to reflect IR solar energy and reduce the emissivity of the surface to significantly enhance the thermal performance. Though perovskites architectures have been reported to reflect IR wavelengths as a “thermal mirror,”^57^ a low-emissivity (low-e) coating of conventional windows also imparts an ultralow emissivity on the interior surfaces of an IGU to minimize radiation heat transfer into the building. Our work combines the optical color-balancing properties of the Bragg reflector we developed with the thermal performance of a low-e coating.

We have effectively designed a low-e layer that selectively reflects red light—a “red low-e.” We employ layers of MoO_3_ (32, 32, and 16 nm, from the glass outward) between 10 nm-thick silver layers and 40 nm SiO_2_ capping layer (Figure 6a). Like commercial triple-silver low-e layers, our red low-e has negligible transmission (high reflection) in the IR region of the spectrum and maximum transmission of ∼0.7 in the visible. However, commercial low-e films are designed to maximize transmission across the LEF, whereas our red low-e strongly reflects red photons and tapers reflectivity toward higher photon energies across the LEF (Figure 6b). As a result, our red low-e laminate layer flattens the transmission spectrum across the LEF when laminated to ultrathin MHP PV devices like the *N* = 2 Bragg reflector described above (Figure 6c). The red low-e layer must be laminated with the photonic crystal stack facing toward the interior of the IGU for high thermal performance, as described above (Table 1).

**Figure 6:**
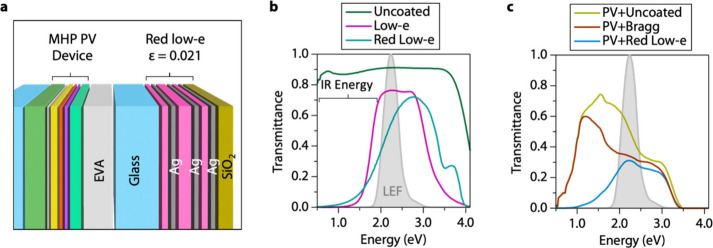
Design of red low-e layer. (a) Diagram of the MHP PV device laminated to a red low-e layer. The EVA layer is 450 μm thick for simulation. Thickness of the MoO_3_ (pink) layers are 32, 32, and 16 nm (from the glass outward). The Ag layers are 10 nm, and the SiO_2_ layer is 40 nm. (b) Transmission spectra of measured uncoated glass (IGDB ID = 5004), triple-silver low-e-coated glass (IGDB ID = 1614) and simulated red low-e-coated glass. IGDB = International Glazing Database. (c) Simulated transmission spectra of an MHP PV device laminated with uncoated glass (PV+Uncoated), Bragg reflector on glass (PV+Bragg), and red low-e on glass (PV+Red Low-e). LEF = luminous efficiency function.

Initial efforts to synthesize red low-e films yielded a neutral gray color when coupled to the device and highly attenuated transmission in the IR as predicted by our model (Figure S8). Unfortunately, the VT of the film was reduced compared to the model, likely due to the ultrathin silver layers dewetting the oxide surface.^58^ Triple and even quad silver low-e films are available commercially, but there are a scarce number of scientific publications on the topic.^59^ Tweaking silver spacing and thickness would allow for optical spectra similar to the red low-e layers presented here by using alternative chemistries or wetting layers that do not lead to the issues found here.

Thermo-optical performance of laminate layers for MHP PV were evaluated using *PVwindow*, coupled to *Optics* and *Window*, which are simulation software packages developed by Lawrence Berkeley National Laboratory.^60^ The software packages are the accepted method for certifying thermal performance metrics reported to U.S. consumers by the National Fenestration Rating Council (NFRC). In *PVwindow*, we simulated the transmission, front reflection, back reflection, and PCE spectra of ultrathin MHP PV devices laminated with uncoated glass, *N* = 2 Bragg reflector on glass, or red low-e on glass. The data was imported into the International Glazing Database (IGDB), where it was read into *Optics* and *Window* (see Methods).

Both software packages allow for the assembly of arbitrary glazing systems with the outboard PV laminate designed in *PVwindow*. While the optical characteristics described in previous sections were modeled for PV laminates without additional lites of an IGU, the thermal attributes of the solar heat gain coefficient and U-factor are strongly dependent on the structure of the IGU and laminates integrated into it. We varied from single glazing (PV laminate only) to double glazing, triple glazing, and VIG (Figure 7a-d). Details on balancing IGU components for each of the glazing systems investigated (coated glass, gas fill, etc.) are outlined in Table S2. Double and triple glazing has lites separated by a 12 mm gap filled with air or argon (Table S2). The VIG has a gap of 0.2 mm vacuum gap. Note that single glazing in this case consists of two layers of glass within the PV laminate, while the double contains three layers of glass total, the triple has four layers, and VIG has three layers of glass. Correspondingly, the surfaces are numbered as 1–4 for the single, 1–6 for the double, 1–8 for the triple, and 1–6 for the VIG using glazing industry convention.

**Figure 7:**
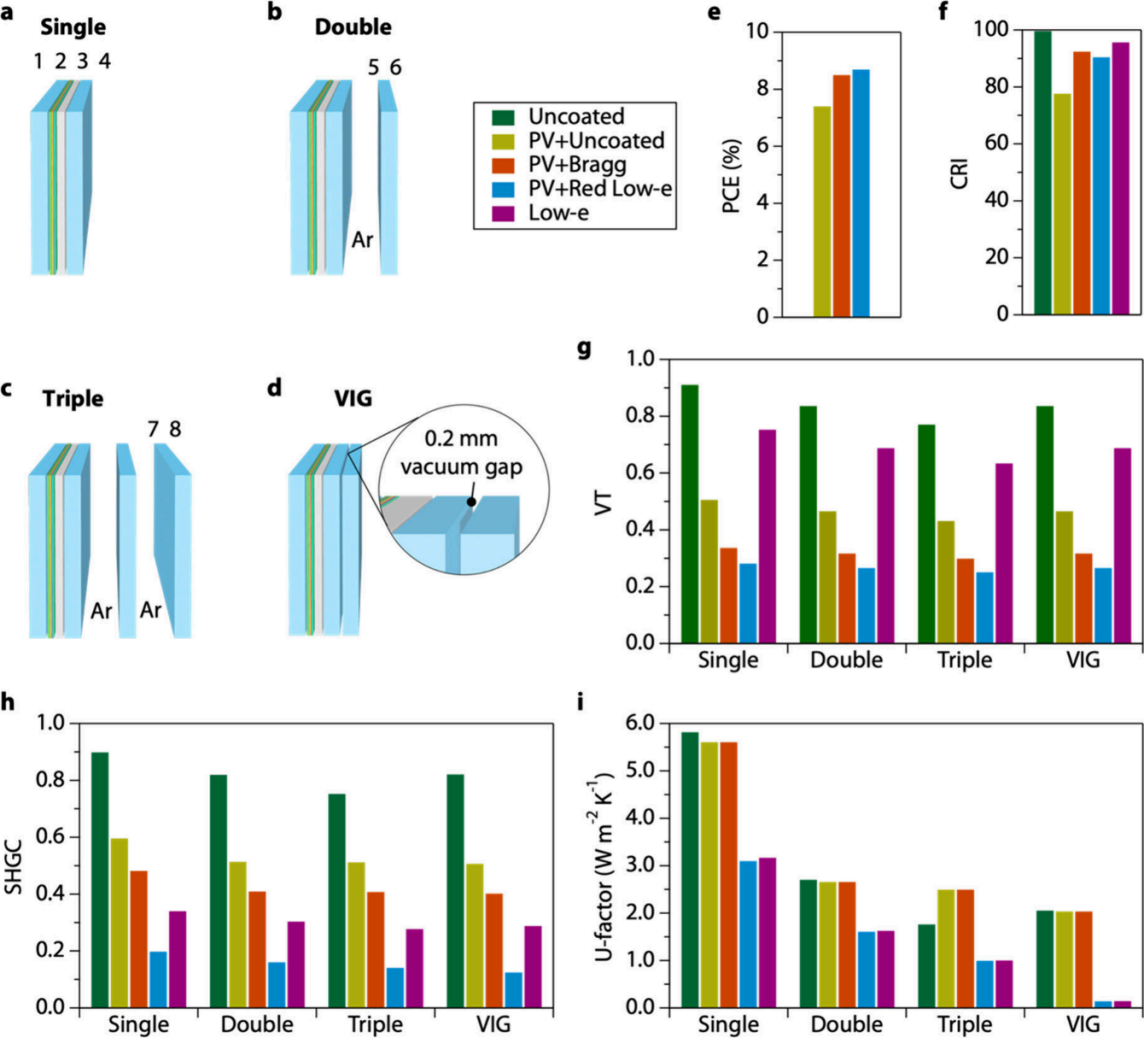
Thermo-optical modeling of MHP PV windows. (a) Diagram of single glazing, (b) double glazing, (c) triple glazing, and (d) VIG. Numbers indicate the number of the glass surface. VIG = vacuum insulating glazing. (e) Power conversion efficiency (PCE), (f) visible transmittance (VT), (g) color rendering index (CRI), (h) solar heat gain coefficient (SHGC), and (i) U-factor for each of the windows studied. Low-e and red low-e layers on surface 4 only for all simulations. (e) and (f) show data for the single configuration since additional lites are not significantly impactful. All data plotted are listed in Table S3.

PCE is unchanged after incorporation of PV laminates into IGUs based on assumptions of the simulation. Ultrathin MHP PV devices laminated with uncoated glass exhibit PCE = 7.4%, whereas laminate layers with Bragg reflectors or red low-e layers exhibit boosted PCE = 8.9% (20.3% increase) and PCE = 8.7% (17.6% increase), respectively (Figure 7e, Table S3). We note that the PCE calculation does not take temperature effects into account. An outboard lite of an IGU will operate at a different temperature compared with a bifacial PV module. A bifacial module is open to convective airflow on both sides of the panel, whereas the IGU-integrated PV will be insulated at the rear of the panel. However, this effect will be offset by fewer photons absorbed and thermalized in a PV window compared with a bifacial module designed to maximize absorption. Additionally, temperature coefficients of MHP PV are highly dependent on device composition, and it is unclear how it will impact results.^61^ We also do not consider illumination from the interior of the building or any secondary reflection from interior lites. Both effects affect each laminate composition in a similar fashion, making the relative comparison between the laminates valid.

Thermo-optical properties of MHP PV IGUs were compared to those of conventional glazing. Specifically, we compared to IGUs of uncoated glass, which were standard in the 80s in 90s and are still the most widespread in the US today, and IGUs of low-e coated glass, which meet current standards and represent the majority of sales today.^62^ Regardless of the outboard laminate, CRI does not vary significantly after IGU incorporation, and the single glazing value is quite accurate for all configurations (Figure 7f, Table S3). Each glazing system varies by no more than 1.4 between single, double, triple, and VIG configurations. The CRI of uncoated low-iron glass is CRI = 99.7. It drops to CRI = 95.6 for commercial low-e and 90.5 for the red low-e layers designed here. VT, however, varies significantly with each additional lite for uncoated glazing. Single glazing is reduced from VT = 0.91 to VT = 0.77 for triple glazing (Figure 7g, Table S3). In contrast, VT was affected less by the addition of extra lites for other configurations. For instance, red low-e laminate layers vary from VT = 0.28 to VT = 0.25 (triple), and low-e (non-PV) IGUs vary from VT = 0.73 to VT = 0.69 (triple).

SHGC varies significantly as a function of outboard lite transmission, since that is the dominant term in SHGC. In fact, since the visible portion of the solar spectrum is the richest in energy, SHGC largely trends with VT for uncoated glass, MHP PV laminated with uncoated glass, and MHP PV laminated with Bragg reflectors (Figure 7h). The exceptions are low-e and MHP PV laminated with red low-e because they target reflection in the IR. The overall lowest SHGC is MHP PV laminated with red low-e because it targets IR and has lower VT than a conventional low-e. Though comparable VT, the red low-e layer ((SHGC = 0.198) is less than half that of the Bragg reflector (SHGC = 0.482). A secondary contribution to SHGC is the energy absorbed and transmitted into the building expressed as *n*(*E*)*I*(E)*A*(*E*) (Table 1). The SHGC for each IGU decreased with the number of lites (single through triple) due to decreased transmission and decreased *n*(*E*). It should be noted that a low SHGC is desired for hot climates and a higher SHGC is beneficial in cold climates for passive solar heating. Future low-e designs could increase IR transmission while maintaining a low emissivity, which is typically achieved with “passive” low-e layers composed of doped oxide coatings instead of triple silver stacks.

High U-factors are favored for all climate zones. U-factor values are lowest for VIG, a technology on the verge of more widespread market accessibility, followed by triple, double, and single glazing. U-factor trends with more gaps (*k*) and is minimized by reducing convective (*h*_g_) and radiative heat transfer coefficient (*h*_*r*_). *h*_g_ is reduced by minimizing the gap conductivity, and *h*_*r*_ is reduced by minimizing the emissivity of the surface (Table 1). It is clear both terms must be reduced to minimize U-factor. For example, double glazing with low-e layers (conventional and red low-e) exhibit a U-factor = 1.3 W m^−2^ K^−1^, whereas triple glazing without low-e layers has a lower *h*_g_ but higher U-factor (U-factor = 1.6 W m^−2^ K^−1^ for uncoated glass, and MHP PV laminated with Bragg reflector). Combining low-e layers with VIG leads to the lowest U-factor with and without PV exhibiting U-factor = 0.14. Superstrate MHP PV modules could be laminated directly to VIG units to produce superinsulating PV windows. Overall, the combination of a PV module with low-e glass and Argon gaps leads to thermally efficient glazing that meets U.S. Energy Star benchmarks of 0.25–0.40 for SHGC and 1.53–2.27 W m^–2^ K^–1^ for U-factor, depending on the region.

A consequence of high U-factors in PV IGUs is a change in the thermal environment that the PV materials experience. Harsh thermal environments could be a particular concern for MHP PV windows since MHP materials have reactive interfaces and exhibit low formation energies,^63^ which makes long-term durability challenging.^64^ We studied the operational temperature of MHP PV windows using the same thermal simulation approach used to determine U-factor (NFRC-100-2010). We expect temperatures of the interior glass surface of the outboard lite will be higher than when integrated into an IGU rather than a typical rack-mounted PV deployment, which is similar to single glazing. PV integration into superinsulating VIG units leads to the most dramatic change in the thermal environment. For a non-PV low-e single glazing in hot summer conditions (32 °C outside air temperature, 24 °C inside air temperature), the inner surface of the glass will be 38.3 °C, whereas the same glass integrated into VIG will be 40.6 °C (Figure S9). In contrast, the inner surface of a single-glazing PV+red low-e laminate will reach 46.4 and 50.6 °C when integrated into a VIG. The operating temperature of PV layers can increase by 10 °C when in a superinsulating IGU, but this is not an alarming temperature for MHP PV durability. MHP PV has demonstrated significant improvements in durability in recent years. Similar device stacks to the one used here have lasted >1000 h at 85 °C and 85% RH while retaining 98.9% of their original efficiency based on PV testing standards (ISOS-D-3).^65^

Photovoltaic glazing has the potential to transform the way we implement glass in buildings by dramatically reducing building energy use and carbon emissions. However, PV windows must satisfy several thermo-optical design criteria in addition to photovoltaic energy conversion, including PCE, VT, LUE, Haze, color, SHGC, and U-factor. In this work, we break down each of these metrics, which need to balance the solar spectrum for optical properties and power conversion as well as control all modes of heat transfer within PV IGUs. We demonstrated a method to fabricate ultrathin (<40 nm), pinhole free MHP thin films and utilize them in a package designed to minimize parasitic absorption. Using experiment and custom TMM software, we design layers that are laminated to ultrathin MHP devices by simulating transmission, reflection, and PCE spectra. Layers consist of one-dimensional photonic crystals designed to achieve neutral color and enhance photocurrent by reflecting transmitted photons back to the absorber. Our experimentally validated photonic crystal was composed of alternating layers of MoO_3_ and MgF_2_ to form a Bragg reflector that selectively reflects red wavelengths, which neutralizes the color of ultrathin MHP devices. We demonstrate experimental MHP devices with VT > 0.35, CRI > 90, and PCE > 5% that match our simulation results. We also simulated a “red low-e” photonic crystal that reflects red photons using thin silver films to decrease the emissivity of the film, which significantly enhances thermal properties when integrated into an IGU. We couple simulated optical and spectral PCE data with software that allows IGU design and thermal performance evaluation. Our holistically designed MHP PV laminate featuring a red low-e layer VIG configuration exhibits superinsulating properties with SHGC = 0.125 and U-factor = 0.144 W m^–2^ K^–1^ while maintaining VT > 0.25, PCE > 8.5%, CRI > 90. We believe this work will inspire more holistic design into next-generation PV window research where thermal properties are emphasized along with power conversion and optical properties.

## Methods

### Materials

Formamidinium iodide >99.99% (FAI), methylammonium bromide >99.99% (MABr) were purchased from Greatcell Solar Materials. PbI_2_, and PbBr_2_ were purchased from TCI. [2-(9H-Carbazol-9-yl)ethyl]phosphonic acid (2PACz) > 98.0% was purchased from Tokyo Chemical Industry (TCI). Fullerene-C60 > 99.9%/>99.5% was purchased from Luminescence Technology Corp. (Lumtec). Bisphenol A propoxylate diglycidyl, pentaerythritol tetrakis (3-mercaptopropiante), and 2,4,6-Tris(dimethylaminomethyl)phenol were purchased from Sigma-Aldrich. All chemicals were used as received without further purification.

### Perovskite Solar Cell Fabrication

Initially, 25 × 25 × 1.1 mm indium-doped-tin-oxide (FTO) patterned substrates were purchased from Colorado Concept Coatings LLC. The substrates were submerged and sonicated for 15 min sequentially in Liquinox diluted in DI water, DI water, acetone, and IPA, followed by 15 min of UV-ozone from a commercial UVO cleaner (Jelight 342). 2PACz, dissolved in ethanol in a 1 mM solution, was statically dispensed onto a substrate, then spun at 3000 rpm for 30 s (1500 rpm/s), and annealed at 100 °C on a hot plate for 10 min. For perovskite films, 15.4 mg CsI, 22.4 mg MABr, 73.4 mg PbBr_2_, 172 mg FAI, and 507 mg PbI_2_ were dissolved in 1 mL of 9:1 DMF:NMP to form the perovskite precursor solution. 80 μL of solution was statically dispensed onto the patterned substrate, and spun at 2000 rpm for 10 s, and 6000 rpm for 30 s. During the spin process, nitrogen was dispensed onto the spinning substrate with 20 s remaining for 10 s and then spun for an additional 10 s using an automated system through a steel straw. Films were transferred immediately to a hot plate and annealed at 100 °C for 30 min.

Fifteen nm C60 was thermally evaporated at 0.2 Å/s for 5 nm and then 0.5 Å/s for the next 10 nm using an Angstrom evaporation system at 4e-7 Torr. Fifteen nm SnO_x_ was deposited via atomic layer deposition (recipe). 80 nm indium zinc oxide (IZO) was sputtered at 100 W for 15 min.

### Bragg Reflector Fabrication

One mm-thick glass substrates were submerged and sonicated for 15 min sequentially in Liquinox diluted in DI water, DI water, acetone, and IPA. Onto the glass MoO_3_ was thermally evaporated at an initial rate of 0.5 Å/s for 10 nm then ramped to 2.0 Å/s. MgF_2_ was electron-beam evaporated at an initial rate of 0.5 Å/s for 10 nm and then ramped to 2.0 Å/s. Both were evaporated using an Angstrom evaporate system with 4e-7 Torr vacuum.

### Lamination Procedure

An in-house epoxy was used to laminate the Bragg reflector to the PV device. Bisphenol A propoxylate diglycidyl (epoxy) was mixed with pentaerythritol tetrakis (3-mercaptopropiante) (thiol) in a 1:2 mol ratio. Right before lamination, a drop of 2,4,6-Tris(dimethylaminomethyl)phenol (initiator) was added, mixed, and placed onto the Bragg reflector. The Bragg reflector was sandwiched on top of the PV device and left to cure overnight. Off-the-shelf epoxies can also be used.

### Solar Cell Characterization

A Sunbrick AAA LED solar simulator was used to measure the PV performance of our devices. The spectrum was calibrated to 1-sun AM1.5G illumination with a Si reference cell and KG2 filter certified by NREL’s certification and measurement group. All devices were measured using a Keithley 2400 source meter under a sweep mode of reverse scan (1.2 V to −0.2 V) and a forward scan (from −0.2 to 1.2 V). The device area by sputtering was 0.12 cm^2^, and the devices were masked with aperture masks of 0.059 cm^2^ during current–voltage scans for proper current-density measurements.

### Electron Microscopy

Samples for STEM analysis was prepared on a Tescan Solaris Ga FIB using the lift-out technique to extract a lamella containing the device cross-section. Tungsten was deposited on the surface to mitigate damage from the incident Ga^+^ ions during preparation. STEM analysis was carried out on a ThermoFisher Scientific Spectra 200 STEM instrument operating at 200 kV and equipped with a Super-X EDS system for EDS imaging.

### Optical Characterization

Optical transmission spectra were obtained by measuring solar cell samples masked with the same aperture masks of 0.059 cm^2^ as those used for solar cell characterization. All six pixels were averaged in the measurement. Samples were placed at the front port of a custom-built integrating sphere system leveraging Ocean Optics Maya 2000 Pro and NIRQuest+2.5 spectrometers to enable measurements from 250 to 2500 nm. Haze measurements were performed on Cary 7000 UV–vis-NIR spectrophotometer outfitted with a diffuse reflectance accessory. Measurements were obtained with a circular aperture masking a single pixel at the front of the sphere with a reflection standard or light trap at the back of the sphere, following from ASTM D1003-21.^23^

### Thermo-Optical Simulation

The simulation methodology is also detailed in our previous report.^7^ We combine our *PVwindow* code to simulate photovoltaic and optical properties with *Optics* and *Window* from Lawrence Berkeley National Laboratory to solve the heat equation and yield the thermal performance of IGUs. The optical and heat transfer models of *Optics* and *Window* require transmission, front reflection, and back reflection spectra. We first simulate transmission, front reflection, back reflection, and spectral efficiency of the encapsulated PV laminate using TMM in *PVwindow*. Transmitted energy affects SHGC, and only absorbed energy affects the U-factor and SHGC by increasing the steady-state temperature of the PV laminate. Since reflection does not contribute to thermo-optical properties of the PV IGU, we add spectral efficiency to the front-side reflection spectrum to ensure converted energy is not included in the absorptance spectrum. It is a way to include thermalization losses in the PV to the energy balance solution but not the energy from PV conversion. We use the same spectral power conversion efficiency equation as in Table 1, which is effectively an EQE spectrum weighted by spectral irradiance (e.g., Figure 5b). We substitute EQE with *A*_MHP_IQE, where *A*_MHP_ is the absorptance of the absorber MHP layer extracted from the TMM calculation. We use *A*_MHP_ to determine the number of absorbed photons in a detailed balance calculation that yields *J*_SC_ as a function of photon energy, *V*_OC_, FF, and spectral PCE. IQE = 1 for all simulations in this work. Different laminates created with transmission, spectral PCE-modified front reflectance, and back reflectance spectral data are imported into the international glazing database (IGDB) in *Optics* software, where CRI is calculated. Simulated laminates in the IGDB were then imported into *Window* where IGUs are built using the IGU components outlined in Table S2. Spacing thicknesses are kept constant at 12 mm for all configurations except VIG, which is reduced to the conventional thickness of commercial VIG units. The PV laminate (with uncoated glass, Bragg layers on glass, or red low-e layers on glass) is placed as the first lite with further standardized IGU layers ordered behind it as indicated in Figure 7. *Window* simulations are performed according to ISO 15099 to yield VT, SHGC, and U-factor. *h*_e_ is taken as 26 W m^–2^ K^–1^ and *h*_i_ as 7.8 W m^–2^ K^–1^ within the National Fenestration Rating Council (NFRC) convention (NFRC-100–2010).

## Supporting Information Available

Luminous efficiency function, CIE standard observer color matching functions, profilometry measurements, absorption coefficients, transmission spectra, transmission spectrum and photographs of experimental red low-e films, sources for complex refractive index data used in transfer matrix method calculations, components for simulated PV IGUs, PV IGU metrics presented in the main text, inner surface temperature of the IGU as a function of outboard lite type (PDF)
