# Supplementary material for: Holistic Thermo-Optical Design of Laminate Layers for Halide Perovskite Photovoltaic Windows
Source: ACS Energy Lett. 2024 Nov 11;9(12):5836–49. doi: 10.1021/acsenergylett.4c02017 (PMC11650772; doi:10.1021/acsenergylett.4c02017)
Supplement: Supplementary file 1 — nz4c02017_si_001.pdf [file nz4c02017_si_001.pdf]

Supplementary Information:

Holistic Thermo-Optical Design of Laminate Layers for Halide Perovskite Photovoltaic Windows

Kevin J. Prince[1,2], Nicholas P. Irvin[1], Mirzo Mirzokarimov[1], Bryan A. Rosales[1], David T. Moore[1], Harvey L. Guthrey,[1] Axel F. Palmstrom[1], Colin A. Wolden,[1,2] and Lance M. Wheeler[1,*]

[1]National Renewable Energy Laboratory, Golden, CO 80401, USA
[2]Department Chemical and Biological Engineering, Colorado School of Mines, Golden, CO 80401, USA

*Correspondence: Lance.Wheeler@nrel.gov

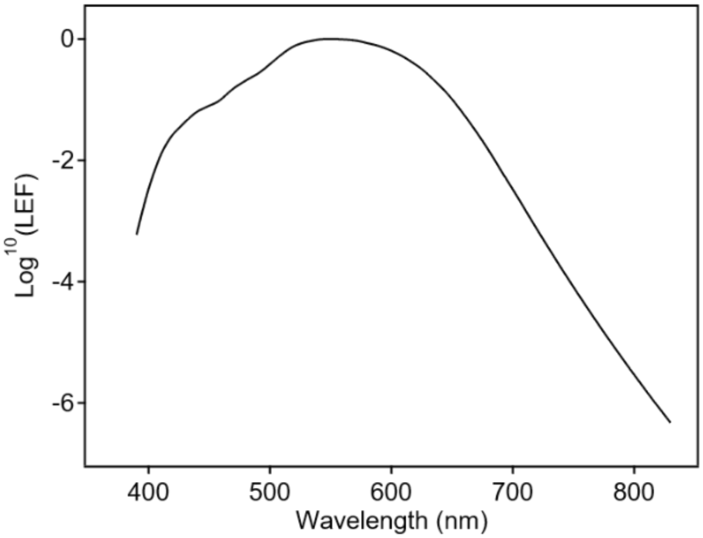

Figure S1. Luminous Efficiency Function (LEF) in (a) linear-linear and (b) log-linear plots.

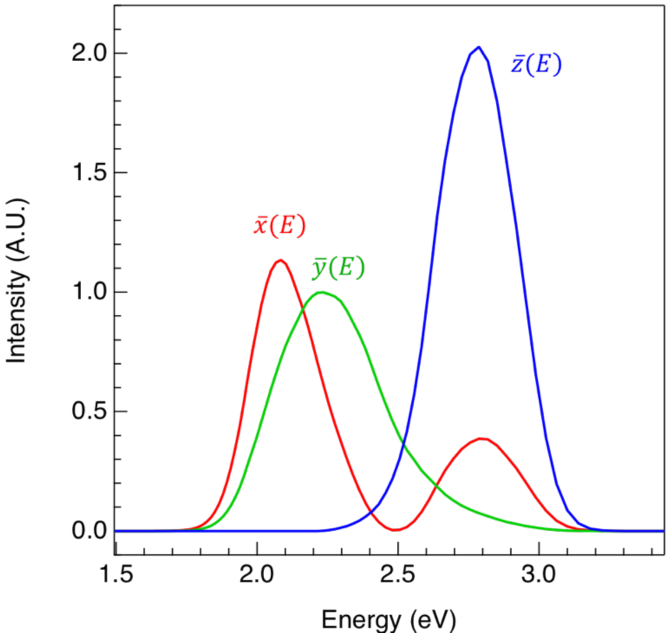

Figure S2. CIE Standard observer color matching functions.

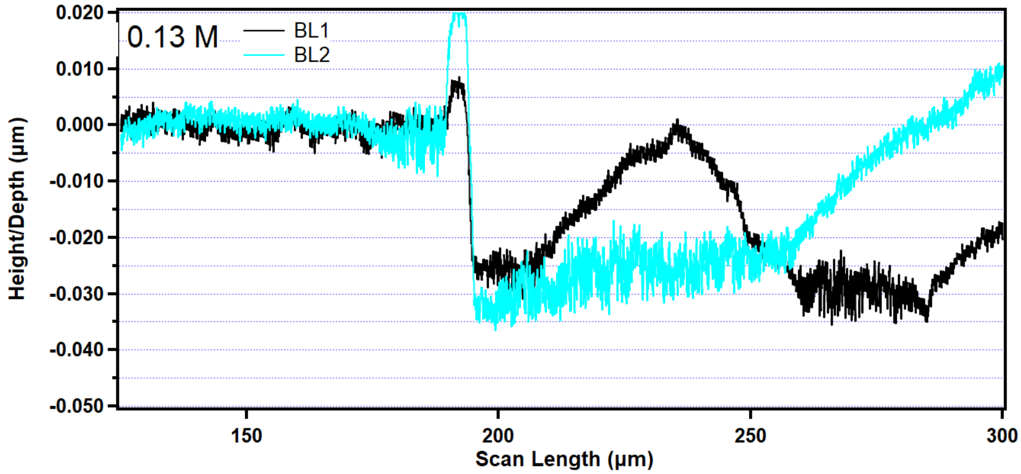

Figure S3. Profilometry measurement of step edge of thinnest halide perovskite film formed from a 0.135 M precursor solution.

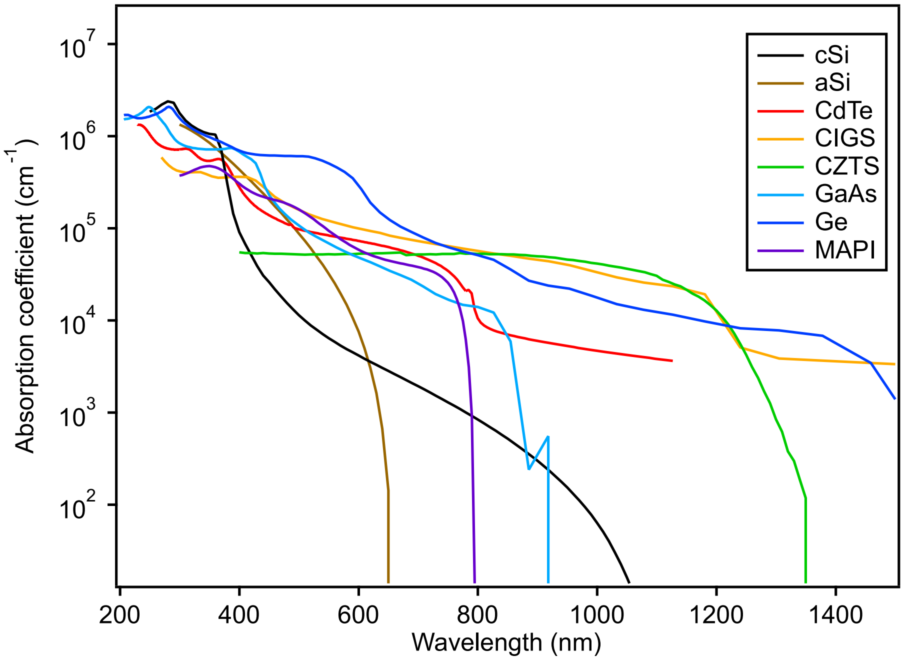

Figure S4. Absorption coefficients of common non-selective semiconductor PV materials including crystalline silicon (cSi), amorphous silicon (aSi), cadmium telluride (CdTe), copper indium gallium selenide (CIGS), copper zinc tin sulfide (CZTS), gallium arsenide (GaAs), germanium (Ge), and methyl-ammonium lead iodide (MAPI).

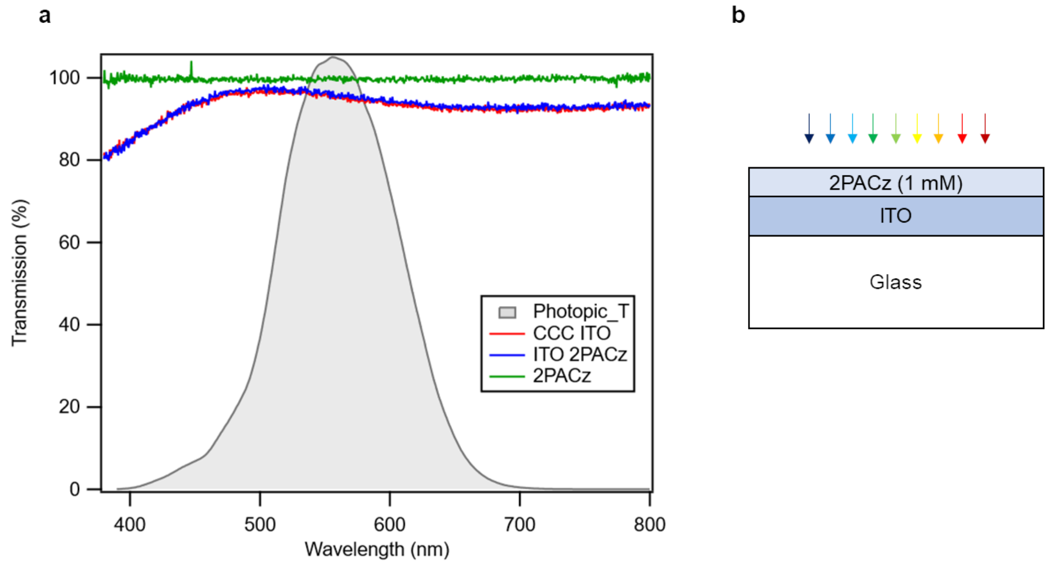

Figure S5. (a) Transmission spectra of Colorado Concept Coatings ITO-glass (red curve), 2PACz coated ITO glass (blue curve) and 2PACZ (green) over the photopic eye response. (b) Schematic of films of transmission spectra.

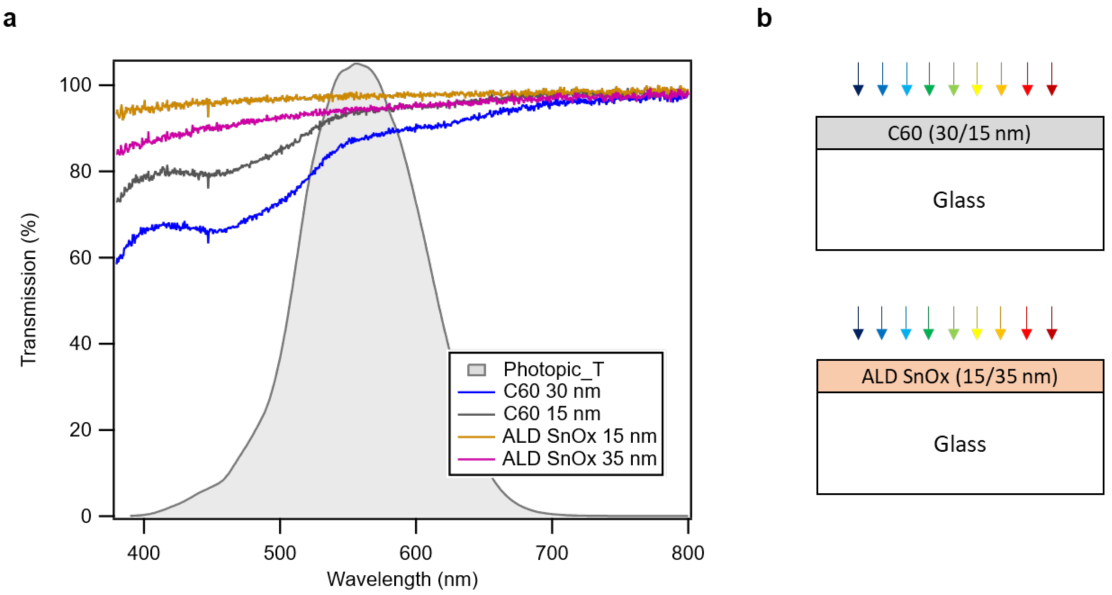

Figure S6. (a) Transmission spectra of varying thicknesses of evaporated C_{60} and atomic layer deposition (ALD) SnO_x. (b) Schematic of films of transmission spectra.

a

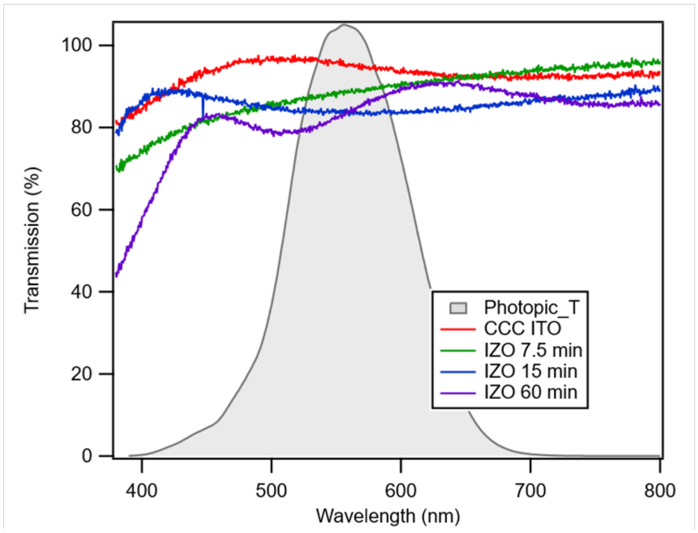

b

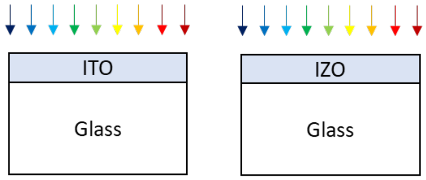

c

IZO sputter time	Sheet Resistance (Ω/□)
7.5 min	150.5 ± 18.0
15 min	70.5 ± 13.6
60 min	12.3

Figure S7. (a) Transmission spectra of varying sputter times and thicknesses of sputtered indium zinc oxide (IZO). (b) Schematic of films of transmission spectra. (c) Sheet resistance measurements of IZO films from 4-point probe measurements.

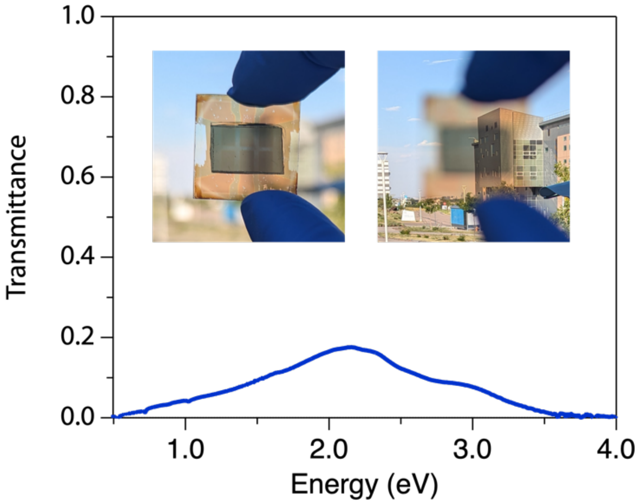

Figure S8. Transmission spectrum and photographs of experimental red low-e films. The photographs focus on the device (left) and a building in the distance (right) to highlight lack of haze. The lack of transmittance is likely due to de-wetting of the silver films, but the properties of the film yield a neutral-colored MHP device.

Table S1: Sources for complex refractive index data used in transfer matrix method calculations. When available, data was used from https://refractiveindex.info/. Data not available was digitized using (https://automeris.io/WebPlotDigitizer/) from the references in the table below. When data was not available from 300 to 2500 nm, the Kramers-Kronig relation was applied to generate the missing data.

Material	Reference
Glass	M. Rubin. Optical properties of soda lime silica glasses, *Sol. Energy Mater.* **12**, 275-288 (1985)
EVA	M. R. Vogt, H. Schulte-Huxel, D. Hinken, H. Holst, M. Winter, S. Blankemeyer, R. Witteck, M. Köntges, K. Bothe, R. Brendel. Optical constants of UV transparent EVA and the impact on the PV module output power under realistic illumination, *Energy Procedia* **92**, 523-530 (2016)
ITO	Alonso-Álvarez, D., Ferre Llin, L., Mellor, A., Paul, D. J. & Ekins-Daukes, N. J. ITO and AZO films for low emissivity coatings in hybrid photovoltaic-thermal applications. *Solar Energy* **155**, 82–92 (2017).

Table S2: Components for simulated PV IGUs. Glass conductivity for each lite is assumed to be 1.0 W m^{-1} K^{-1}. The surface emissivity of other surfaces IGU is 0.84 for bare glass. Glass conductivity for each standard is taken at 1.0 W m^{-1} K^{-1}. "*PVwindow*" indicates the optical properties were simulated and imported to a local database.

		**Lite 1**					**Lite 2**			**Lite 3**
**Window Type**	**IGU design**	**IGDB ID**	**Thickness** (mm)	**Surface 4 emissivity**	**Gas fill**	**Gap distance** (mm)	**IDGB ID**	**Gas fill**	**Gap distance** (mm)	**IDGB ID**
Uncoated	Single	5004	5.7	0.84	-	-	5004	-	-	-
Uncoated	double	5004	5.7	0.84	Air	12	5004	-	-	-
Uncoated	Triple	5004	5.7	0.84	Argon	12	5004	Argon	12	5004
Uncoated	VIG	5004	5.7	0.84	0.0001 Pa air	0.2	5004	-	-	-
Low-e	Single	1614	6	0.021	-	-	-	-	-	-
Low-e	double	1614	6	0.021	Argon	12	5004	-	-	-
Low-e	Triple	1614	6	0.021	Argon	12	5004	Argon	12	5004
Low-e	VIG	1614	6	0.021	0.0001 Pa air	0.2	5004	-		-
PV + Uncoated	Single	*PVwindow*	12	0.84	-	-	-	-	-	-
	double	*PVwindow*	12	0.84	Argon	12	5004	-	-	-
	Triple	*PVwindow*	12	0.84	Argon	12	5004	Argon	12	5004
	VIG	*PVwindow*	12	0.84	0.0001 Pa air	0.2	5004	-		-<br>-
PV + Bragg	Single	*PVwindow*	12	0.84	-	-	-	-	-	-
	double	*PVwindow*	12	0.84	Argon	12	5004	-	-	-
	Triple	*PVwindow*	12	0.84	Argon	12	5004	Argon	12	5004
	VIG	*PVwindow*	12	0.84	0.0001 Pa air	0.2	5004	-	-	-
PV + Red Low-e	Single	*PVwindow*	12	0.021	-	-	-	-	-	-
	double	*PVwindow*	12	0.021	Argon	12	5004	-	-	-
	Triple	*PVwindow*	12	0.021	Argon	12	5004	Argon	12	5004
	VIG	*PVwindow*	12	0.021	0.0001 Pa air	0.2	5004	-	-	-

Table S3: PV IGU metrics presented in the main text. All values are for center-of-glass calculations.

		PV Metrics				Optical Metrics								Thermal Metrics	
**Window Type**	**IGU design**	**PCE**	**Voc** (V)	**Jsc** (mA cm^{-2})	**FF**	**VT**	**T Color (Window)** L*	a*	b*	**Rf Color (Window)** L*	a*	b*	**CRI**	**SHGC**	**U-factor** (W m^{-2} K^{-1})
Uncoated	Single	-	-	-	-	0.911	96.45	-0.23	0.10	34.41	-0.15	-0.71	99.651	0.899	5.820
Uncoated	double	-	-	-	-	0.836	93.26	-0.44	0.18	45.72	-0.30	-0.82	99.431	0.820	2.704
Uncoated	Triple	-	-	-	-	0.771	90.36	-0.65	-0.65	52.80	-0.46	-0.87	99.175	0.754	1.620
Uncoated	VIG	-	-	-	-	0.836	93.26	-0.44	0.18	45.72	-0.30	-0.82	99.431	0.821	2.057
Low-e	Single	-	-	-	-	0.753	86.39	-3.74	2.87	41.60	-1.22	-7.51	95.637	0.340	3.171
Low-e	double	-	-	-	-	0.688	86.39	-3.74	2.87	41.60	-1.22	-7.51	95.637	0.300	1.315
Low-e	Triple	-	-	-	-	0.634	83.60	-3.81	2.77	47.19	-2.11	-5.84	95.100	0.278	1.002
Low-e	VIG	-	-	-	-	0.688	86.39	-3.74	2.87	41.60	-1.22	-7.51	95.637	0.288	0.145
PV + Uncoated	Single	0.074	1.349	6.051	0.907	0.506	75.67	9.19	21.52	61.28	-14.20	-3.84	77.674	0.596	5.608
	double	0.074	1.349	6.051	0.907	0.466	73.23	8.44	20.75	63.06	-12.30	-1.98	78.405	0.514	2.497
	Triple	0.074	1.349	6.051	0.907	0.432	71.00	7.78	20.06	64.52	-10.90	-0.50	79.076	0.461	1.605
	VIG	0.074	1.349	6.051	0.907	0.466	73.23	8.44	20.75	63.06	-12.30	-1.98	77.674	0.507	2.034
PV + Bragg	Single	0.089	1.343	7.274	0.906	0.337	64.48	1.12	6.93	70.33	-2.40	11.36	93.044	0.482	5.608
	double	0.089	1.343	7.274	0.906	0.317	62.87	0.99	7.49	70.99	-2.31	11.51	93.044	0.408	2.497
	Triple	0.089	1.343	7.274	0.906	0.299	61.34	0.85	7.93	71.57	-2.23	11.66	92.832	0.367	1.605
	VIG	0.089	1.343	7.274	0.906	0.317	62.87	0.99	7.49	70.99	-2.31	11.51	92.437	0.402	2.034
PV + Red Low-e	Single	0.087	1.338	7.100	0.906	0.282	59.84	-4.38	6.08	69.64	-0.05	9.54	90.470	0.198	3.099
	double	0.087	1.338	7.100	0.906	0.266	58.35	-4.19	6.68	70.11	-0.20	9.66	90.733	0.152	1.301
	Triple	0.087	1.338	7.100	0.906	0.251	56.94	-4.03	7.17	70.53	-0.32	9.77	90.904	0.141	0.995
	VIG	0.087	1.338	7.100	0.906	0.266	58.35	-4.19	6.68	70.11	-0.20	9.66	90.470	0.125	0.144

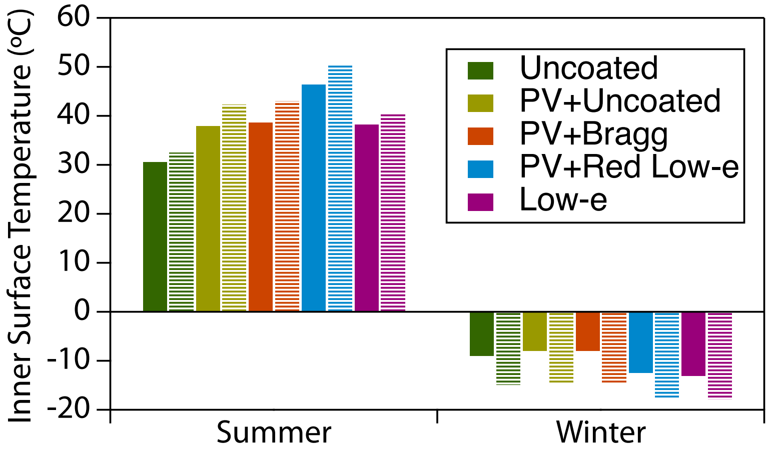

Figure S9. Inner surface temperature of the IGU as a function of outboard lite type. Temperature is reported for surface 2 for uncoated and Low-e glass and surface 4 for PV laminates. Solid bars represent single glazing whereas dashed bars indicate VIG.
